# A novel *Geobacteraceae*-specific outer membrane protein J (OmpJ) is essential for electron transport to Fe (III) and Mn (IV) oxides in *Geobacter sulfurreducens*

**DOI:** 10.1186/1471-2180-5-41

**Published:** 2005-07-06

**Authors:** Eman Afkar, Gemma Reguera, Marianne Schiffer, Derek R Lovley

**Affiliations:** 1 Department of Microbiology, University of Massachusetts, Amherst, Massachusetts 01003, USA; 2 Biosciences Division, Argonne National Laboratory, Argonne, Illinois 60439, USA

## Abstract

**Background:**

Metal reduction is thought to take place at or near the bacterial outer membrane and, thus, outer membrane proteins in the model dissimilatory metal-reducing organism *Geobacter sulfurreducens *are of interest to understand the mechanisms of Fe(III) reduction in the *Geobacter *species that are the predominant Fe(III) reducers in many environments. Previous studies have implicated periplasmic and outer membrane cytochromes in electron transfer to metals. Here we show that the most abundant outer membrane protein of *G. sulfurreducens*, OmpJ, is not a cytochrome yet it is required for metal respiration.

**Results:**

When outer membrane proteins of *G. sulfurreducens *were separated via SDS-PAGE, one protein, designated OmpJ (outer membrane protein J), was particularly abundant. The encoding gene, which was identified from mass spectrometry analysis of peptide fragments, is present in other *Geobacteraceae*, but not in organisms outside this family. The predicted localization and structure of the OmpJ protein suggested that it was a porin. Deletion of the *ompJ *gene in *G. sulfurreducens *produced a strain that grew as well as the wild-type strain with fumarate as the electron acceptor but could not grow with metals, such as soluble or insoluble Fe (III) and insoluble Mn (IV) oxide, as the electron acceptor. The heme *c *content in the mutant strain was ca. 50% of the wild-type and there was a widespread loss of multiple cytochromes from soluble and membrane fractions. Transmission electron microscopy analyses of mutant cells revealed an unusually enlarged periplasm, which is likely to trigger extracytoplasmic stress response mechanisms leading to the degradation of periplasmic and/or outer membrane proteins, such as cytochromes, required for metal reduction. Thus, the loss of the capacity for extracellular electron transport in the mutant could be due to the missing *c*-type cytochromes, or some more direct, but as yet unknown, role of OmpJ in metal reduction.

**Conclusion:**

OmpJ is a putative porin found in the outer membrane of the model metal reducer *G. sulfurreducens *that is required for respiration of extracellular electron acceptors such as soluble and insoluble metals. The effect of OmpJ in extracellular electron transfer is indirect, as OmpJ is required to keep the integrity of the periplasmic space necessary for proper folding and functioning of periplasmic and outer membrane electron transport components. The exclusive presence of *ompJ *in members of the *Geobacteraceae *family as well as its role in metal reduction suggest that the *ompJ *sequence may be useful in tracking the growth or activity of *Geobacteraceae *in sedimentary environments.

## Background

Fe (III) oxide is the most abundant metal electron acceptor in most soils and sediments and its microbial reduction greatly contributes to the degradation of organic matter in many sedimentary environments as well as to the degradation of organic contaminants in polluted groundwater [1,2]. Fe (III) also is the primary electron acceptor supporting the growth of dissimilatory metal-reducing microorganisms when metal reduction is stimulated by adding a suitable electron donor to promote the *in situ *bioremediation of soluble metal contaminants [3] such as uranium [4] and vanadium [5]. Thus, understanding the mechanisms of electron transfer to insoluble Fe (III) oxides could greatly aid in the study of dissimilatory metal reduction in various sedimentary environments.

Despite the wide phylogenetic diversity of microorganisms capable of dissimilatory metal-reduction [1], molecular analyses of moderate temperature sedimentary environments in which Fe (III) reduction is important have routinely found that microorganisms in the *Geobacteraceae *are prevalent whereas other well-studied Fe (III)-reducing microorganisms, such as *Shewanella *species, are not detected [1]. This has been attributed, at least in part, to different mechanisms for Fe (III) reduction in these organisms. *Geobacter *species need to directly contact Fe (III) oxides in order to reduce them [6], have a highly specialized strategy for searching for Fe (III) oxides [7], and use pili as conductive nanowires to transfer electrons to the insoluble electron acceptor [8]. In contrast, *Shewanella *[9,10] and *Geothrix *species [11] produce soluble electron shuttles and Fe(III) chelators which alleviate the need for direct contact with Fe (III) oxides.

Because of the insoluble nature of Fe(III) and Mn(VI) oxides, metal reduction in dissimilatory metal-reducing organisms is thought to occur at or near the outer membrane. Most studies on the mechanisms for Fe (III) reduction in *Geobacter *species have focused on the role of *c*-type cytochromes [12-14]. Over 110 putative *c*-type cytochrome genes have been identified in the *G. sulfurreducens *genome [15]. Many of these cytochrome genes are more highly expressed during growth on Fe (III) than with fumarate as the electron acceptor and deletion of some of these cytochrome genes greatly reduces the capacity for Fe (III) reduction [13,14]. However, the importance of *c*-type cytochromes in the final electron transfer to Fe (III) has been questioned because *Pelobacter *species, which are phylogenetically intertwined with *Geobacter *and *Desulfuromonas *species in the *Geobacteraceae *[16], can reduce Fe (III), yet do not appear to contain *c*-type cytochromes [17]. If, as expected, reduction of Fe(III) takes place at, or near, the outer membrane surface, then there may be outer membrane proteins other than *c*-type cytochromes, which have a role in electron transfer to Fe(III). In support of this, *Geobacter*'s pili have recently been found to play a key role in electron transfer to insoluble metals by acting as microbial nanowires that extend the electron transfer capabilities of the bacterium beyond the cell surface [8].

*Geobacter sulfurreducens *has been routinely used as a model organism for investigations into Fe (III) reduction in *Geobacteraceae *because its complete genome sequence [15] and a genetic system [18] are available. Here we report that the most abundant protein in the outer membrane of *G. sulfurreducens *is not a cytochrome, yet this protein is required for Fe (III) reduction.

## Results

### Identification and characterization of OmpJ

**Figure 1:**
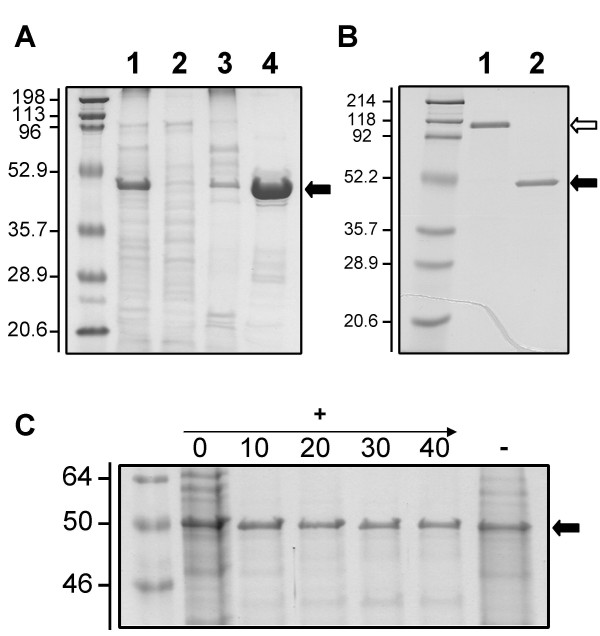
**Localization and characterization of OmpJ. A: **OmpJ is the most abundant protein in the outer membrane of *G. sulfurreducens*. Subcellular fractions (cell-free extracts (lane 1), soluble (lane 2), cytoplasmic membrane (lane 3) and outer membrane (lane 4) fractions; 5 μg protein per lane), of *G. sulfurreducens *grown with fumarate as electron acceptor were analyzed by SDS-PAGE. The most abundant protein in the outer membrane (indicated by an arrow) was designated OmpJ. **B: **Heat modifiability of OmpJ. OmpJ migrated as a dimer (white arrow) in an SDS-PAGE gel in the absence of heat treatment (lane 1) but migrated as a monomer (solid arrow) after heat treatment at 100°C for 5 minutes (lane 2). Protein, 5 μg per lane. **C: **Effect of proteinase K treatment on OmpJ integrity. Outer membrane fractions were treated with different concentrations (0 to 40 U ml^-1^) of proteinase K (+) to analyze the surface exposure of OmpJ and their protein composition was analyzed by denaturing PAGE. A negative control without proteinase K (-) also is shown. First lane in all panels corresponds to molecular weight standards. Numbers at left are molecular masses in kDa.

One protein, designated outer membrane protein J (OmpJ), was much more abundant than any other protein in the outer membrane fraction of cells grown with fumarate as the sole electron acceptor (Fig. 1A). This protein also was present in cultures grown with other electron acceptors such as Fe(III) citrate, Fe(III) oxides and and Mn(IV) oxides (data not shown). MALDI-TOF mass spectrometry analyses identified eight peptides (MGDATVALGFAR, VDFGGWAANATAK, LITHFEIDSTWGK, FDPVTIDGFLLYQR, NVYLDENIPSTPLNVK, AFAIANVGFVAADKDNTTYCNAR, ALVYNVQNVIGGFVGYNANITSK, VFDNLTASVQGAYVILGDYFKDTAGTAANPEDPR) that uniquely corresponded to the protein encoded by ORF GSU3304 (gi-39998393), annotated as a putative LamB porin family protein in the genome of *G. sulfurreducens*. OmpJ had a predicted average molecular mass of 48.9 kDa, in accordance with its mobility in denaturing gels (Fig. 1A), and a theoretical pI of 6.25 compared with a pI of 6.7 determined by isoelectric focusing gel electrophoresis. The PSORT algorithm predicted OmpJ to be localized in the outer membrane, consistent with its presence in outer-membrane preparations (Fig. 1A).

Several secondary structure prediction methods predicted that OmpJ consists mainly of extended beta-chain fragments. Six of the first 20 predictions by 3D-PSSM [19], which uses a threading algorithm to predict the fold of a protein most homologous to structures deposited in the Protein Data Bank, suggest that OmpJ is a porin. The other predicted folds were for soluble proteins, which have large numbers of beta segments and thus appear to be inappropriate for a membrane-bound protein.

Several pieces of biochemical evidence also indicate that OmpJ may function as a porin. First, its localization in the outer membrane. OmpJ was associated to the outer membrane fraction of *G. sulfurreducens *and was isolated in a highly purified form after removal of the cytoplasmic membrane (Fig. 1A). Also, the apparent molecular weight of OmpJ in the absence of heating (118 kDa) was roughly twice that of the heat-treated protein (Fig. 1B), indicative of heat modifiability [20,21]. In addition, *in situ *proteinase K treatment of intact cells of *G. sulfurreducens *did not digest OmpJ (Fig. 1C) while OmpJ integrity was affected after proteinase K treatment of outer membrane fractions (data not shown). Incubation of intact cells with proteinase K leads to the degradation of exposed outer membrane proteins, while subsurface proteins such as porins remain protected against proteolysis. Taken together, these data greatly strengthen our assertion that (i) OmpJ is located in the outer membrane, (ii) it is tightly embedded within the membrane and (iii) it is not significantly exposed on the cell surface, as expected of a porin.

### Presence of *ompJ *in other Geobacteraceae

**Figure 2:**
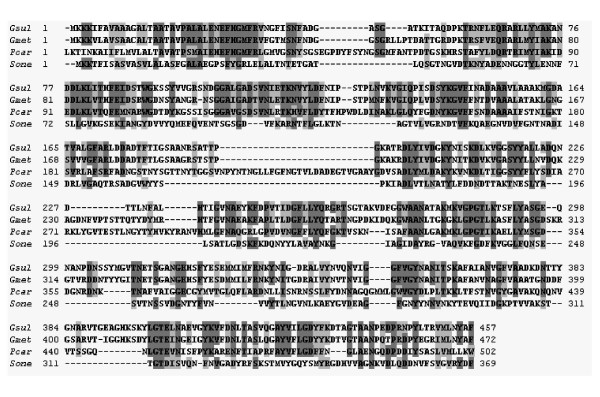
**Amino acid sequence alignment of OmpJ, OmpJ homologs in other members of the *Geobacteraceae *and Omp35, a porin-like protein from *S. oneidensis *MR-1. **Identical residues are highlighted in dark grey and similar residues in light grey. Abbreviations: Gsul; *Geobacter sulfurreducens *OmpJ; Gmet, *G. metallireducens *OmpJ homolog; Pcar, *P. carbinolicus *OmpJ homolog; Sone, *S. oneidensis *MR-1 Omp35.

Homologs of *ompJ *were found in the genomes of the two other members of the *Geobacteraceae *for which complete sequences are available,*Geobacter metallireducens *and *Pelobacter carbinolicus *(Fig. 2). A hypothetical protein in the genome of *G. metallireducens *(gi-48845525) had the highest identity at the amino acid level, 70.2% (out of 476 amino acids), and 80% similarity to OmpJ of *G. sulfurreducens*. A protein also was identified in *P. carbinolicus *(GenBank, accession number DQ004247) that shared 34% identity (out of 513 amino acids) and 51% similarity with *G. sulfurreducens *OmpJ. No close OmpJ homologs were found in the NCBI database outside the *Geobacteraceae *family. OmpJ had 21% identity and 33.3% similarity at the amino acid level with Omp35 of *S. oneidensis *MR-1 (Fig. 2), a porin-like protein found to have an indirect effect in metal reduction in this organism [22]. However, while OmpJ had no similarity to other porins in the database, Omp35 was most closely similar to known bacterial porins such as PorA of *Neisseria meningitidis *[22]. These results suggest that OmpJ is a novel type of porin that is unique to the *Geobactereaceae*.

### Characterization of an OmpJ-deficient mutant

**Figure 3:**
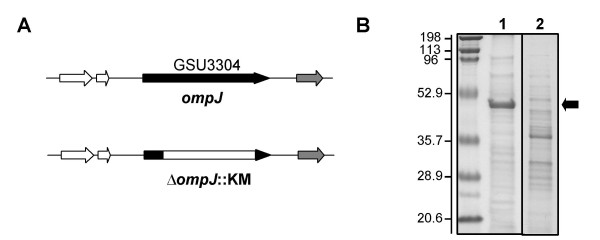
**Generation of an OmpJ-deficient mutant of *G. sulfurreducens*. A: **An OmpJ-deficient mutant of *G. sulfurreducens *was constructed by gene replacement with a kanamycin cassette. **B: **Protein composition of outer membrane fractions of the wild-type (lane 1) and an OmpJ-deficient mutant (lane 2) showing the absence of the OmpJ band in the mutant strain (indicated by an arrow). First lane corresponds to molecular weight standards. Numbers at left are molecular masses in kDa.

The presence of the *ompJ *gene in the *Geobacteraceae *family but not in any other group of bacteria, suggested that OmpJ might play a key role in the physiology of these metal reducers. In order to elucidate the physiological role of *G. sulfurreducens *OmpJ, a deletion mutant was constructed by gene replacement with a kanamycin cassette (Fig. 3A). SDS-PAGE analyses confirmed the absence of the OmpJ protein band in outer membrane preparations of the OmpJ-deficient mutant but also showed significant differences in the protein composition of the mutant outer membrane fraction when compared to the wild-type, with some absent proteins and some new proteins present in the outer membrane fractions of the mutant (Fig. 3B).

**Figure 4:**
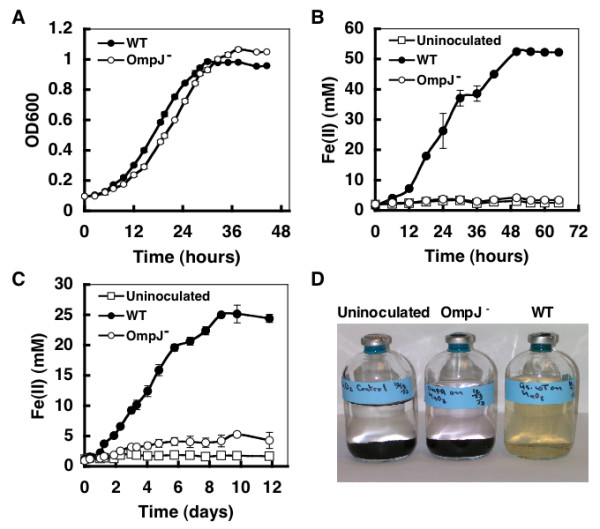
**Physiological characterization of an OmpJ-deficient mutant. **The growth of the wild-type (WT) and OmpJ-deficient mutant (OmpJ-) strains with fumarate (**A**), Fe(III) citrate (**B**), and insoluble Fe(III) (**C**) or Mn(VI) (**D**) oxides was studied. Growth on fumarate was quantified by following the optical density of the culture at 600 nm (OD600), while growth with soluble or insoluble Fe(III) was monitored by measuring the levels of soluble Fe(II) present in the medium as a result of Fe(III) reduction. Error bars are the standard deviation from the average of triplicate determinations. The reduction of Mn(VI) oxides was visually tested as the oxidized Mn(VI) oxides turn from black to a whitish precipitate upon reduction. Uninoculated controls also were included in **B–D**.

The OmpJ-deficient mutant strain reduced fumarate nearly as well as the wild-type strain (Fig. 4A). However, it was markedly deficient in the reduction of metals such as soluble Fe (III) citrate (Fig. 4B), insoluble poorly crystalline Fe (III) oxide (Fig. 4C), as well as Mn (IV) oxides (Fig. 4D) when compared to the wild-type strain. Attempts to complement the mutation *in trans *yielded a strain that produced OmpJ only at levels ca. 10% of the wild-type, as visualized on denaturing gels (data not shown). Such suboptimal levels of complementation have previously been observed in previous studies on the function of other genes in *G. sulfurreducens *[13,14]. However, even with suboptimal levels of OmpJ, 16% of the Fe (III) citrate reduction capacity was restored (data not shown).

**Figure 5:**
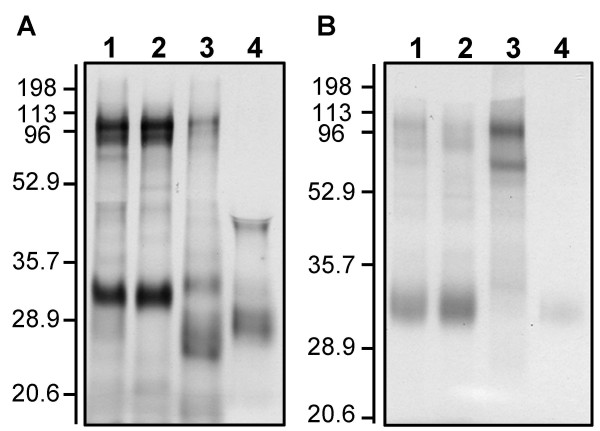
**Heme-stained protein composition of a WT (A) and an OmpJ-deficient mutant (B). A: **Distribution of heme-stained proteins in subcellular fractions of *G. sulfurreduces *wild-type. Lane 1, total cell free extract; lane 2, soluble fractions; lane 3, inner membrane; and lane 4, outer membrane; 10 μg protein per lane. **B: **Distribution of heme-stained proteins in subcellular fractions of an OmpJ-deficient mutant. Lane 1, total cell free extract; lane 2, soluble fractions; lane 3, inner membrane; and lane 4, outer membrane; 10 μg protein per lane. Numbers at left are molecular masses in kDa.

The loss of the capacity for Fe (III) and Mn (IV) reduction in the *ompJ *mutant was associated with a significant loss of *c*-type cytochromes from the cell. The *c*-type cytochrome content in cell extracts of the wild-type was 20 ± 0.7 μmol per 100 mg protein whereas the mutant's *c*-type cytochrome content was 10 ± 0.2 μmol per 100 mg protein. Heme *c *staining of proteins separated by SDS-PAGE confirmed there was a loss of a number of both soluble and membrane-associated cytochromes in the OmpJ-deficient mutant strain (Fig. 5). There was also an apparent increase in the abundance of a 70 kDa *c*-type cytochrome associated with the inner membrane.

**Figure 6:**
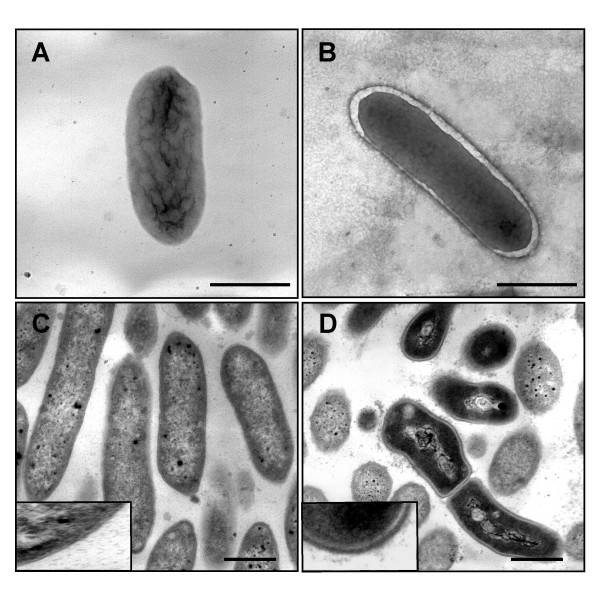
**Microscopic analyses of an OmpJ-deficient mutant. **Transmission Electron Microscopy analyses of cells of the wild-type (**A **and **C**) and an OmpJ-deficient mutant (**B **and **D**) showing the enlargement of the periplasmic space in the OmpJ-mutant cells when compared with the wild type. **A **and **B **show negatively stained cells while **C **and **D **show micrographs of thin sections of, respectively, cells of the wild-type and OmpJ-deficient mutant strains. Bars, 1 μm.

Because porins also have been shown to play a structural role in the integrity of the bacterial cell surface [23,24], cells were examined with transmission electron microscopy (TEM). TEM analyses of negatively-stained cells and of thin sections of cells from fumarate-grown cultures showed that the appearance of the mutant cells was remarkably different from the wild-type cells and was consistent with an enlarged periplasm (Fig. 6).

## Discussion

The results presented in this work suggest that the most abundant outer-membrane protein in *G. sulfurreducens *is a probable porin and, despite its apparent lack of any moieties capable of participating in electron transfer, its presence is required in order for *G. sulfurreducens *to reduce extracellular electron acceptors, such as soluble Fe(III) citrate and insoluble Fe (III) and Mn (IV) oxides. These results, coupled with the recent discovery of the importance of a porin-like protein, designated Omp35, in Fe (III) reduction in *Shewanella oneidensis *MR-1 [22], emphasize that porins play an important role in the maintenance of the physical integrity and function of the cell surface in dissimilatory metal-reducing microorganisms.

### Physiological role of OmpJ

OmpJ's annotation as a porin is consistent with the predicted beta-barrel structure of the protein [25] and with several pieces of biochemical evidence such as its location and abundance in the outer membrane (Fig. 1A) and the results of our proteinase K study, which suggest that it is embedded in the membrane (Fig. 1C). Also, porins often assemble in the outer membrane as multimeric structures composed of several porin monomers [24] and, as a result, many porins exhibit heat modifiability [20,21]. As was shown in Fig. 1B, OmpJ also showed heat modifiability.

Bacterial outer membrane porins have been studied extensively [24,25] and porin-like proteins also are found in chloroplasts and mitochondria [26]. Porins represent an unusual class of membrane proteins in that they exhibit no hydrophobic stretches in their amino acid sequences and form hollow beta-barrel structures with a hydrophobic outer surface. The barrel structure encompasses the trans-membranous pore that allows the passive diffusion of hydrophilic solutes across the (outer) membrane [25]. The enlarged periplasm observed in the OmpJ-deficient mutant, as compared to that of the wild type cells, further suggests some role in solute transport. Although OmpJ is annotated in the genome of *G. sulfurreducens *as a putative protein of the LamB porin superfamily, this classification does not necessarily imply a physiological role analogous to that of LamB channels. The LamB superfamily comprises channels involved in the spontaneous diffusion of specific nutrients. The LamB protein of *E. coli*, which also serves as the phage lambda receptor, is a prototype of this class of channels and catalyzes the influx of maltose and maltodextrins and also facilitates the influx of various carbohydrates when in low concentrations in the extracellular environment [24]. However, *Geobacter *species are not known to use sugars as substrates. The finding that the *ompJ *deletion mutant reduced fumarate at rates comparable to the wild-type suggests that the transport of the electron donor, acetate, or essential nutrients, such as ammonia, phosphate, sulfur, amino acids and trace metals, which are necessary in central metabolic reactions, was not inhibited. Morevover, the enlarged periplasmic space observed upon deletion of the *ompJ *gene is consistent with a channel involved in the efflux, rather than influx, of some nutrient.

It is unlikely that OmpJ is involved in the final electron transfer to Fe (III) and Mn (IV) because it lacks any apparent electron transfer moieties and because it appears to be embedded within the membrane rather than exposed externally. In addition, other non-cytochrome proteins, type IV pili, have recently been shown to be involved in the final electron transfer to insoluble metals [8]. Thus, the effect of the *ompJ *mutation in the inhibition of Fe (III) and Mn (IV) reduction appears to be indirect, as a result of the general reduction in the production of *c*-type cytochromes. Previous studies have suggested that a number of *c*-type cytochromes are required in order for *G. sulfurreducens *to effectively reduce metals and the loss of just one of these may inhibit Fe (III) reduction [13,14,27]. In some instances, outer-membrane proteins may be required for the proper localization of outer-membrane *c*-type cytochromes [28] and, if not properly localized and folded, cytochromes may be proteolytically degraded. However, a direct role for OmpJ in the proper localization of *c*-cytochromes is unlikely because OmpJ homologs are also present in *Pelobacter *species, which reduce Fe (III) but do not contain detectable *c*-type cytochromes [17]. Alternatively, OmpJ may be required for transport of some constituent such as iron that is required for *c*-cytochrome production. The swelling of the periplasm in fumarate-grown cells suggests, however, that transport of one or more solutes out of the cell might be inhibited in the absence of OmpJ. This abnormal periplasm is likely to interfere with protein folding and localization, thus inducing the extracytoplasmic stress responses, mediated by the RpoE sigma factor and Cpx systems in *E. coli *[29], and triggering a proteolytic cascade that relieves the accumulation of misfolded proteins in the periplasm [30].

### Porins and metal reduction

OmpJ of *G. sulfurreducens *is the first porin ever to be described in detail in any member of the delta-Proteobacteria [24]. Similar to Omp35, a porin-like protein from *Shewanella oneidensis *MR-1 [22], it appears to have an indirect role in metal reduction. While an Omp35-deficient mutant in *S. oneidensis *had slower rates of reduction of fumarate and soluble or insoluble Fe(III) [22], deletion of OmpJ of *G. sulfurreducens *did not affect fumarate reduction but dramatically inhibited metal reduction such as soluble or insoluble Fe(III) and Mn(VI) oxides. This is not surprising inasmuch as fumarate reduction occurs intracellularly in *G. sulfurreducens *[31] while the *S. oneidensis *terminal reductase is periplasmic [32]. Interestingly, the levels and distribution of key components of electron transport in *S. oneidensis *such as quinones and cytochromes were normal in the Omp35-deficient mutant [22], whereas the OmpJ-deficient mutant of *G. sulfurreducens *had a substantial reduction in heme *c *and *c*-cytochrome abundance, which may have indirectly contributed to the marked decrease of its metal reduction potential. The lack of homology and differences in function between Omp35 and OmpJ may reflect the profound differences in the mechanisms for Fe(III) reduction in *Shewanella *and *Geobacter *species [1].

Most importantly, these studies emphasize the role of non-electron transport proteins in electron transfer to a variety of electron acceptors. OmpJ of *G. sulfurreducens *is of special significance because, unlike Omp35, it is widespread in members of the *Geobacteraceae*, but no homologs are found in any other bacterial groups. This suggests that screening for *ompJ*-like sequences may be a good strategy for identifying *Geobacteraceae *sequences in libraries of environmental genomic DNA. Furthermore, *ompJ *provides another gene in the short, but growing, list of *Geobacteraceae*-specific sequences that might be used to quantify the number of *Geobacteraceae *in Fe (III)-reducing environments or to infer rates of activity of these organisms from quantitative mRNA analysis [33,34].

## Conclusion

In summary, OmpJ is an abundant outer-membrane protein in *G. sulfurreducens *and it is required for metal reduction in this organism. OmpJ also is required to keep the structural integrity of the periplasmic space, which is necessary for proper folding and functioning of electron transport components. Thus, the role of OmpJ in metal reduction may, in fact, be indirect. While the actual role of this apparent porin is still uncertain, further studies on interactions of OmpJ with other proteins in *G. sulfurreducens *may help to better elucidate its function.

## Methods

### Bacterial strains and culture conditions

All strains used in this work were isogenic with the wild-type *G. sufurreducens *strain PCA (ATCC 51573) [35], obtained from our laboratory culture collection. A deletion mutant in the *ompJ *gene (GSU3304) was constructed by cross-over PCR replacing the +311 to +1245 coding region with a kanamycin (KM) cassette, as previously described [27,36]. Briefly, the upstream region of *ompJ *was amplified with primers upF (5'-GCGTTGACAGACAAACTC-3') and upR (5'-GCCATCGTTCGATCTTCCG-3') and the downstream region with primers dwnF (5'-CAAGGTGTTTGACAACCTG-3') and dwnR (5'- CAAGGTGTTTGACAACCTG-3'). The KM-resistance cassette in plasmid pBBR1MCS-2 [37] was PCR-amplified with primers kanF (5'- CGGAAGATCGAACGATGGCACCTGGGATGAATGTCAGC-3') and kanR (5'-CAGGTTGTCAAACACCTTGATGGCAGGTTGGGCGTCGC-3'). The three PCR-amplified fragments were used as templates in a recombinant PCR reaction [38] using primers upF and dwnR to amplify a 1.951 kb DNA fragment. Electroporation and mutant isolation were performed as described previously [18]. The mutation was confirmed by PCR and Southern blotting [38]. The *ompJ *mutation was complemented *in trans *using plasmid pCM66-OmpJ, a pCM66 [39] derivative carrying a wild-type copy of the *ompJ *coding region previously amplified using primers OmpJ01 (5'-GGAAGCTTCCATGCTGTTTTATCATACCC-3') and OmpJ02 (5'-GGGAATTCGGTGATGCAATTAGAATG-3').

Cells were routinely cultured under strict anaerobic conditions in freshwater (FW) medium, as previously described [40], with 20 mM sodium acetate as the electron donor and either 55 mM Fe (III)-citrate, 40 mM fumarate, poorly crystalline Fe (III) oxide (100 mmol/l), or MnO_2 _(3 mol/l) as the electron acceptor.

### Preparation of outer membrane proteins and PAGE analyses

Cells for protein analyses were grown in 1-liter bottles or, for mass cultivation, in 10-liter carboys to late-exponential phase, harvested by centrifugation (12,000 xg for 10 min at 4°C), and washed with 10 mM Tris-HCl (pH 8.0) containing 1 mM EDTA and 10 μM phenol methyl-sulfonyl fluoride (PMSF) to inhibit proteolytic activities. Cell samples were routinely stored at -20°C. Before use, cell samples were suspended in 10 mM Tris-HCl buffer (pH 7.5) at 4°C, and subjected to three passes through a French pressure cell at 10,000 psi to lyze the cells (cell-free extract). Cell debris and intact cells were separated by centrifugation (12,000 g for 20 min) and the cell-free extract was further centrifuged (100,000 × g for 1 h) to separate the soluble fraction (SF) from the crude membrane pellet. The pellet was resuspended in 10 mM Tris-HCl buffer (pH 7.5) and lauroyl sarcosine-sodium salt was added to a final concentration of 1% (w/v) in order to selectively solubilize cytoplasmic membrane proteins [41]. The mixture was stirred at room temperature for 1 to 2 h and then centrifuged at 100,000 × g for 1 h. The supernatant, containing the solubilized cytoplasmic membrane protein fraction (CM), was collected and stored at -20°C. The pellet, which contains the outer membrane fraction (OM), was washed three times in 10 mM Tris-HCl buffer (pH 7.5) containing 10 mM MgCl_2_, 2% NaCl, and 10 μM PMSF.

Protein profiles in the various fractions were analyzed by SDS-Polacrylamide Gel Electrophoresis (SDS-PAGE, 15 %) in a Mini-Protean^® ^3 Cell (Bio-Rad). Prior to electrophoresis, OM protein samples were mixed (1:2) in Laemmli sample buffer (Bio-Rad), containing β-mecaptoethanol and boiled for 10 min at 100°C. Protein separation prior to staining for heme *c*-containing proteins with *N,N,N',N'*-tetramethylbenzidine [42,43] was performed in the same manner except that β-mecaptomethanol was omitted from the sample buffer and boiling time was reduced to 5 min. The most abundant protein band from the OM fraction was excised from the gel, digested with trypsin in the presence of 0.01% n-octylglucopyranoside, and analyzed with matrix-assisted laser desorption ionization- time of flight (MADI-TOF) mass spectrophotometry (Kratos Axima CFR; Kratos Analytical, Manchester, England) [44].

### *In situ *proteinase K treatment

Surface exposure of the OmpJ protein was assayed with proteinase K as a modification of a previously described protocol [45]. Cells were grown to mid-exponential phase in FW medium containing 20 mM acetate and 40 mM fumarate and harvested by centrifugation (20 min at 13,000 rpm, 4°C). After two washes in a 10 mM Tris-HCl buffer (pH 8.0) containing 10 mM MgCl and 2% NaCl, cells were suspended in the same buffer to a final concentration of 63 mg of wet cells per ml. Examination of the bacterial suspension by phase-contrast microscopy did not indicate detectable bacterial lysis. Proteinase K was gradually added to the cell suspension at concentrations ranging from 0 to 40 U ml^-1^. A negative control with added buffer, instead of proteinase K, and another using outer membrane fractions, instead of intact cells, also were included. Samples were stirred at room temperature for 15 min with various concentrations (0 to 40 U ml^-1^) of proteinase K before a protease cocktail inhibitor (Roche) was added to stop the proteolytic reaction. The cells were pelleted by centrifugation at 14,000 rpm for 5 min, washed twice in 10X volumes of the same buffer containing the protease inhibitor cocktail, and resuspended in the same buffer with the protease inhibitor cocktail. Aliquots of the cell suspensions (10 mg protein ml^-1^) were diluted 1:2 in SDS-sample buffer and boiled for 10 min prior to separation by SDS-PAGE, as described above.

### *C*-type cytochromes analyses

Cells of the wild-type and mutant strains were grown to mid-exponential phase with fumarate as the electron acceptor and harvested. Cell-free extract, soluble, CM, and OM protein fractions were prepared as described above. The total heme *c *content of each fraction (100 μg protein) was determined by the pyridine ferrohemochrome method [46]. In addition, every fraction (10 μg protein) was separated by SDS- PAGE (15 %) at 125 V for 2–3 hrs and the gel was stained for heme *c*-containing proteins as previously described [42,43].

### Transmission Electron Microscopy (TEM)

Cells for TEM analyses were grown to mid-exponential phase in FW medium with fumarate, fixed on to the surface of carbon-coated copper grids with glutaraldehyde and negatively stained with 2% uranyl acetate. For thin sections, cells were fixed in glutaraldehyde and stained with osmium tetraoxide following standard microscopy procedures. Samples were analyzed in a JEOL 100S transmission electron microscope operated at 60–80 V.

### Analytical methods

Fe(II) production was monitored by the ferrozine assay at 12 hr intervals [40]. Protein concentration was determined by the bicinchoninic acid method with Bovine Serum Albumin (BSA) as a standard [47].

### Sequence analyses

The PSORT algorithm  was used to predict the cell localization of the OmpJ protein. The genome sequences of *Geobacter metallireducens *and *Pelobacter carbinolicus *can be found at . The genome sequence of *G. sulfurreducens *can be found at .

## Authors' contributions

EA conducted most of the experiments and analyzed some of the data. GR conducted the microscopy experiments and some phenotypic characterization, analyzed and organized the data, and drafted most of the manuscript. MS conducted the analyses of the OmpJ porin amino acid sequence. DRL conceived the overall project, provided experimental guidance, and drafted portions of the manuscript. All authors read and approved the final manuscript.
